# Association between *IL-18 *gene polymorphisms and biopsy-proven giant cell arteritis

**DOI:** 10.1186/ar2962

**Published:** 2010-03-23

**Authors:** Rogelio J Palomino-Morales, Tomas R Vazquez-Rodriguez, Orlando Torres, Inmaculada C Morado, Santos Castañeda, Jose A Miranda-Filloy, Jose L Callejas-Rubio, Benjamin Fernandez-Gutierrez, Miguel A Gonzalez-Gay, Javier Martin

**Affiliations:** 1 Instituto de Parasitología y Biomedicina Lopez-Neyra, CSIC, Parque Tecnológico de Ciencias de la Salud, Avenida del Conocimiento s/n Armilla, Granada-18100, Spain; 2 Division of Rheumatology, Hospital Xeral-Calde, c/Dr.Ochoa, Lugo 27004, Spain; 3 Rheumatology Service, Hospital Clínico San Carlos, c/Profesor Martín Lagos, S/N Madrid - 28040, Spain; 4 Department of Rheumatology, Hospital de la Princesa, Universidad Autónoma, c/Diego de León 62, Madrid, 28006, Spain; 5 Department of Internal Medicine, Hospital Clínico San Cecílio, Avenida Doctor Olóriz 16 Granada 18012, Spain; 6 Division of Rheumatology, Hospital Universitario Marques de Valdecilla, Santander-39008, Spain

## Abstract

**Introduction:**

The objective was to investigate the potential implication of the *IL18 *gene promoter polymorphisms in the susceptibility to giant-cell arteritis (GCA).

**Methods:**

In total, 212 patients diagnosed with biopsy-proven GCA were included in this study. DNA from patients and matched controls was obtained from peripheral blood. Samples were genotyped for the *IL18*-137 G>C (rs187238), the *IL18*-607 C>A (rs1946518), and the *IL18*-1297 T>C (rs360719) gene polymorphisms with polymerase chain reaction, by using a predesigned TaqMan allele discrimination assay.

**Results:**

No significant association between the *IL18*-137 G>C polymorphism and GCA was found. However, the *IL18 *-607 allele A was significantly increased in GCA patients compared with controls (47.8% versus 40.9% in patients and controls respectively; *P *= 0.02; OR, 1.32; 95% CI, 1.04 to 1.69). It was due to an increased frequency of homozygosity for the *IL18 *-607 A/A genotype in patients with GCA (20.4%) compared with controls (13.4%) (*IL18 *-607 A/A versus *IL18 *-607 A/C plus *IL18 *-607 C/C genotypes: *P *= 0.04; OR, 1.59; 95% CI, 1.02 to 2.46). Also, the *IL18*-1297 allele C was significantly increased in GCA patients (30.7%) compared with controls (23.0%) (*P *= 0.003; OR, 1.48; 95% CI, 1.13 to 1.95). In this regard, an increased susceptibility to GCA was observed in individuals carrying the *IL18*-1297 C/C or the *IL18*-1297 C/T genotypes compared with those carrying the *IL18*-1297 T/T genotype (*IL18*-1297 C/C plus *IL18*-1297 T/C versus *IL18*-1297 T/T genotype in GCA patients compared with controls: *P *= 0.005; OR, 1.61; 95% CI, 1.15 to 2.25). We also found an additive effect of the *IL18 *-1297 and -607 polymorphisms with *TLR4 *Asp299Gly polymorphism. The OR for GCA was 1.95 for combinations of genotypes with one or two risk alleles, whereas carriers of three or more risk alleles have an OR of 3.7.

**Conclusions:**

Our results show for the first time an implication of *IL18 *gene-promoter polymorphisms in the susceptibility to biopsy-proven GCA. In addition, an additive effect between the associated *IL18 *and *TLR4 *genetic variants was observed.

## Introduction

Giant cell, arteritis (GCA) is a large- and medium-sized blood vessel systemic vasculitis characterized by the granulomatous involvement of the aorta and especially its cranial branches [1]. GCA is now considered the most common systemic vasculitis in elderly individuals from Western countries [2,3]. Dendritic cells localized at the adventitia-media border of normal medium-sized arteries play a critical role in the initiation of this vasculitis [4]. The inflammatory activity of vascular lesions in GCA is mediated by adaptive immune responses, with CD4 T cells undergoing clonal expansion in the vessel wall and releasing interferon (IFN)-γ [4]. In the experimental mouse model of GCA, systemic administration of ligands for Toll-like receptor (TLR)2 or TLR4 in human artery-SCID chimeras led to differentiation of adventitial dendritic cells into chemokine-producing effector cells with high-level expression of both CD83 and CD86 and mediated T-cell recruitment through release of interleukin (IL)-18 [4]. GCA is also known to be associated with upregulation of IFN-γ, which is critically involved in modulating the process of intimal hyperplasia, leading to the severe ischemic complications observed in this vasculitis [5]. Interestingly, IFN-γ activity is promoted by IL-18, a proinflammatory cytokine, member of the IL-1 cytokine family, which has been shown to exert innate and acquired immune responses [6,7]. IL-18 is expressed by a wide range of immune cells [8] and can mediate both Th1 and Th2 driven immune responses [9,10]. Of potential implication in GCA, IL-18 in combination with IL-12, induces IFN-γ production in Th1 cells, B cells, and natural killer cells, promoting Th1-type immune responses [11,12]. However, IL-18 may also stimulate Th2 immune responses in the absence of IL-12 [13,14].

GCA is a complex polygenic disease [15]. Besides a strong association of GCA with genes that lie within the major histocompatibility complex (MHC) [16-21], many other studies have shown the implication of genetic variants in key components of immune and inflammatory pathways in GCA susceptibility or clinical expression of this vasculitis [21-34].

*IL18 *gene is located on chromosome 11q22.2-22.3 [35] and several polymorphisms within the *IL18 *promoter gene have been associated with different inflammatory and autoimmune diseases [36-43].

An important step forward in our understanding of the pathogenesis of autoimmune diseases may be to establish the presence of shared mechanisms that may lead to a variety of very different complex autoimmune diseases. Taking all these considerations together, in this study we sought to establish the potential role of three polymorphisms (-137, -607, -1297) within the promoter of the *IL18 *gene in the susceptibility to biopsy-proven GCA.

## Materials and methods

### Patients

In total, 212 patients diagnosed with biopsy-proven GCA and 405 controls were included in this study. All of the patients fulfilled the 1990 American College of Rheumatology criteria for the classification of GCA [44]. Inclusion criteria [45] and clinical features of the patient population were described previously [46]. Also, definitions for specific features of the disease, such as polymyalgia rheumatica (PMR), visual ischemic complications, or other severe ischemic manifestations, have been previously described [47,48]. In all cases, biopsy-proven GCA patients were initially treated with prednisone, 40-60 mg/day, for 3 to 4 weeks. Methyl-prednisolone boluses (1 g daily for 3 days) followed by 60-mg prednisone/day for 3 to 4 weeks were used in most patients who had visual ischemic complications or strokes. The prednisone dose was progressively tapered until discontinuation. Apart from visual complications or strokes that were irreversible in some cases, other typical features of the disease such as headache, asthenia, jaw claudication, or PMR improved after corticosteroid therapy. A decrease of erythrocyte sedimentation rate was observed in all cases after the onset of corticosteroid therapy.

Patients and controls are Caucasians, with at least two previous generations born in the corresponding regions, and were included in this study after written informed consent. We obtained approval for the study from the local ethical committees.

### IL18 polymorphisms selection

Several variations within the *IL18* gene promoter region are responsible for changes in the transcription rate [49,50]. In the present study, we selected two functional *IL18* promoter polymorphisms (*IL18* -137 and -607), which were suggested to alter the *IL18* promoter activity. To investigate further into genetic variants within the *IL18* promoter region, we observed in the database [51] a variant in this region that could have a potential role in IL-18 expression (*IL18*-1297 or rs360719). We also studied this polymorphism based on the minor allele frequency and its ability to bind the transcription factor Oct-1. Location of the polymorphisms site was based on the GenBank Accession Nos. [Genbank:AB015961] and [Genbank:BC007461] as the reference sequence. Interestingly, we recently confirmed that the *IL18-1297* gene polymorphism has a functional association with systemic lupus erythematosus [52].

### IL18 genotyping methods

DNA was obtained from peripheral blood mononuclear cells, by using standard methods. The genotyping of the three *IL18 *polymorphisms was performed by using predesigned TaqMan SNP Genotyping Assays (Applied Biosystems, Foster City, CA), as previously described [52].

### Statistical analysis

We used the χ^2 ^test for Hardy-Weinberg equilibrium and statistical analysis to compare allelic and genotypic distributions. Genotype distribution was assessed by using the χ^2 ^test. Odds ratio (ORs) and 95% confidence intervals (95% CIs) were calculated according to Woolf's method by using the Statcalc program (Epi Info 2002; Centers for Disease Control and Prevention, Atlanta, GA, USA). *P *values < 0.05 were considered statistically significant. LD was calculated by using Haploview v 4.0. A logistic regression model was used to estimate gene-gene interaction between the *IL18 *and *TLR4 *SNPs and for the additive effects of the three SNPs. Fisher's Exact test was used to test for the difference in *IL18 *and *TLR4 *-risk allele counts between Cases and Controls. Logistic regression analyses were performed by using the software STATA (v.10.1).

## Results

### IL18 gene polymorphisms are associated with susceptibility to GCA

The case/control ratio was 1:2, approximately. The estimated power of this study for an estimated OR between 1.5 and 2.0 was 77% to 99.5%.

No evidence of departure from Hardy-Weinberg equilibrium was observed in controls.

Table 1 shows the allele and genotype frequencies of the *IL18 *-137 G>C, -607 C>A, and -1,297 T>C polymorphisms in biopsy-proven GCA patients and healthy subjects.

**Table 1:** *IL18 *gene polymorphisms in a series of biopsy-proven GCA and matched controls

*IL18* Polymorphisms	GCA patients Number (%)	Healthy controls Number (%)		
**-137 (G->C) (rs187238)**	**Number = 212**	**Number = 403**	***P *value**	**OR (95% CI)**

G/G	106 (50.0)	224 (55.6)	Reference	^-^
G/C	94 (44.3)	159 (39.4)	0.20	1.25 (0.87-1.79)
C/C	12 (5.7)	20 (5.0)	0.53	1.27 (0.56-2.84)
G	306 (72.2)	607 (75.3)	Reference	^-^
C	118 (27.8)	199 (24.7)	0.23	1.18 (0.89-1.55)

**-607 (C->A) (rs1946518)^a^**	**Number = 212**	**Number = 405**	***P *value**	**OR (95% CI)**

C/C	53 (24.9)	129 (31.9)	Reference	^-^
C/A	116 (54.7)	220 (54.3)	0.21	1.28 (0.85-1.94)
A/A	43 (20.4)	56 (13.8)	0.02	1.87 (1.09-3.21)
C	221 (52.2)	478 (59.1)	Reference	^-^
A	203 (47.8)	332 (40.9)	0.02	1.32 (1.04-1.69)

**-1297 (T->C) (rs360719)^b^**	**Number = 212**	**Number = 405**	***P *value**	**OR (95% CI)**

T/T	99 (46.7)	237 (58.5)	Reference	^-^
T/C	96 (45.3)	150 (37.0)	0.02	1.53 (1.07-2.20)
C/C	17 (8.0)	18 (4.4)	0.02	2.26 (1.063-4.82)
T	294 (69.3)	624 (77.0)	Reference	^-^
C	130 (30.7)	186 (23.0)	0.003	1.48 (1.13-1.95)

^a^Genotype distribution for the *IL18 *-607 (C->A) polymorphism: *P *= 0.054.

^b^Genotype distribution for the *IL18 *-1297 (T->C) (rs360719): *P *= 0.011.

Genotype frequencies for the *IL18 *-607 (C->A) (rs1946518) polymorphism: *IL18 *-607 A/A homozygous versus *IL18 *-607 C/A plus *IL18 *-607 C/C: *P *= 0.04; OR, 1.59; 95% CI, 1.02-2.46.

Genotype frequencies for the *IL18-*1297 (T->C) (rs360719) polymorphism: *IL18-*1297 C/C plus *IL18-*1297 T/C genotypes compared to *IL18-*1297 T/T genotype: *P *= 0.005; OR, 1.61; 95% CI, 1.15-2.25.

No significant association between the *IL18 *-137 G>C and GCA was observed. However, when the *IL18 *-607 C>A was assessed, we found that the frequency of allele A was significantly increased in biopsy-proven GCA patients compared with controls (47.8% versus 40.9%, respectively; *P *= 0.02; OR, 1.32; 95% CI, 1.04-1.69). It was due to a significantly increased frequency of homozygosity for the *IL18 *-607 A/A genotype in the group of patients with biopsy-proven GCA compared with controls (20.4% versus 13.8 in patients and controls, respectively; *IL18 *-607 A/A homozygous versus *IL18 *-607 C/A plus *IL18 *-607 C/C: *P *= 0.04; OR, 1.59; 95% CI, 1.02-2.46) (Table 1).

Interestingly, a significant association between biopsy-proven GCA and the *IL18*-1297 T>C was also found. In this regard, the *IL18*-1297 allele C frequency was significantly increased in biopsy-proven GCA patients (30.7%) compared with controls (23.0%) (*P *= 0.003; OR, 1.48; 95% CI, 1.13-1.95) (Table 1). Moreover, the genotype distribution of the *IL18*-1297 T>C polymorphism disclosed statistically significant differences between biopsy-proven GCA patients and controls (*P *= 0.011). It was due to a reduced frequency of individuals carrying the *IL18*-1297 T/T genotype in the group of biopsy-proven GCA patients (46.7%) compared with the controls (58.5%). In this regard, an increased susceptibility to GCA was observed in individuals carrying the *IL18*-1297 C/C or the *IL18*-1297 C/T genotypes (*IL18*-1297 C/C) plus T/C genotypes versus T/T genotype in GCA patients compared with controls: *P *= 0.005; OR, 1.61; 95% CI, 1.15-2.25). We did not perform a haplotype analysis because the most associated SNP rs360719 (-1297) is not located in a haplotype block, but is a singleton.

### IL18 gene polymorphisms are not associated with clinical manifestation of GCA patients

In a further step, we stratified GCA patients according to the presence of PMR, visual ischemic complications, and severe ischemic manifestations. However, no significant differences were observed when GCA patients were compared according to the presence or absence of these specific clinical features of the disease (data not shown).

### Additive effects of the IL18 and TLR4 risk alleles in GCA

**Figure 1:**
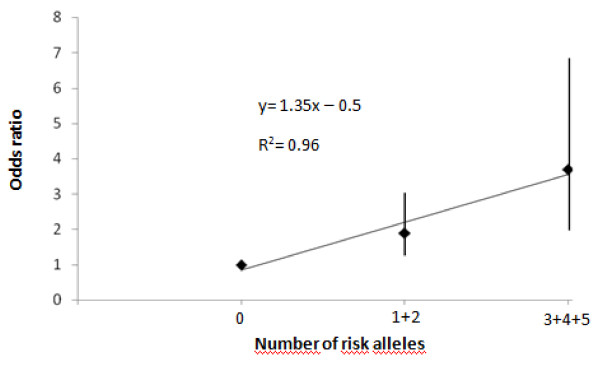
**Combined effects of the risk alleles of (*IL18 *-607 and -1297) and *TLR4 *(Asp299Gly) on susceptibility to GCA**. Linear regression analysis showed an additive effect of the risk alleles of the *IL18 *and *TLR4 *on GCA susceptibility. The ORs with 95% CI are shown as a function of number of risk alleles of GCA.

We recently reported an association between the *TLR4 *Asp299Gly polymorphism and GCA in our population [46]. In the present study, we investigated the potential combined effect of the risk *IL18 *and *TLR4 *alleles on GCA susceptibility by using an additive logistic regression model. The distribution of the different combinations of *IL18 *and *TLR4 *risk alleles in GCA patients and controls is shown in Additional file 1. The overall difference in risk allele counts between GCA patients and controls was statistical significance, *P *= 0.01. We observed an additive effect of risk alleles on susceptibility to GCA. Figure 1 shows the OR for GCA according to the presence of one or two and three or more risk alleles among these three genetic variants, by using the individuals with zero risk allele as the reference group. As shown in the figure, the risk of GCA increases as a function of the number of risk alleles, in an additive manner. Thus, the OR for GCA is 1.9 (CI, 1.1-2.3) for carriers of one or two risk alleles, and 3.7 (CI, 1.9-7.2) for carriers of three or more risk alleles.

## Discussion

In the present study, we examined for the first time the contribution of three polymorphisms in the promoter region of the *IL18 *gene for the susceptibility to GCA. Our results support a potential role of the *IL18 *-607 C>A (rs1946518) and the *IL18*-1297 T>C (rs360719) gene polymorphisms in the predisposition to biopsy-proven GCA. Individuals carrying the *IL18 *-607 A/A showed an increased risk of having GCA compared with controls. A protective effect against the development of GCA was found in individuals carrying the *IL18*-1297 T/T genotype. In contrast, an increased risk of GCA was observed in individuals carrying the *IL18*- 1297 allele C.

Proinflammatory cytokines play a major role in the pathogenesis of GCA [53], a disease associated with a high inflammatory response [54]. IL-18 is a proinflammatory cytokine that induces T-helper 1 differentiation and has cytotoxic T-lymphocyte functions. IL-18 has also emerged as a pivotal cytokine in different autoimmune diseases [55]. A number of functional polymorphisms within the proximal promoter of the *IL18 *gene that may interfere with transcription-factor-binding sites have been verified [49,50]. The implication of the *IL18 *-1297 T>C polymorphism in the susceptibility to GCA also has functional relevance because recent data from our group confirmed that the relative quantification of mRNA performed in total RNA from 23 healthy individuals carrying different genotypes for *IL18 *-1297 T>C (rs360719) polymorphism was associated with an increased expression in individuals carrying the C allele (CC+CT versus TT) [52]. Interestingly, Nabili *et al*. [56] reported and increased expression of IL18 in temporal artery biopsies of GCA patients, with no correlation with clinical manifestations or hematologic parameters. All these data are in accordance with our results and support a potential role of these gene variants in the susceptibility to GCA but not in the phenotypic expression of this vasculitis.

It has been proposed that a variety of inflammatory and autoimmune diseases may share common pathogenic mechanisms. *IL18 *promoter gene polymorphisms have been associated with several autoimmune diseases. With respect to this, an association of the *IL18 *-137 G>C [rs187238] but not the *IL18 *-607 C>A (rs1946518) gene polymorphism with susceptibility to type I diabetes was reported in a study [39]. However, another study of the same two promoter polymorphisms in patients with type I diabetes showed an increased frequency of *IL18 *-607 CA genotype compared with control subjects, but no significant difference in the *IL18 *-137 allele frequencies [57]. No significant association was found when the *IL18 *-137 G>C (rs187238) and the *IL18 *-607 C>A (rs1946518) gene polymorphisms were studied in patients with multiple sclerosis, Crohn disease, or ulcerative colitis [50,58].

In keeping with the results derived from a study on Spanish individuals diagnosed with rheumatoid arthritis (RA) [59], in the present study, we did not find a significant association between the *IL18 *-137 (rs187238) polymorphism and biopsy-proven GCA.

A protective effect mediated by the *IL18 *-607 A/A genotype was observed in Asian patients with RA [43]. It was not the case for Spanish individuals with RA [59]. However, according to our results, an association exists between biopsy-proven GCA and the *IL18 *-607 (rs1946518) gene polymorphism. Moreover, our data show an additional association of biopsy-proven GCA with *IL18 *-1297 T>C (rs360719).

Taken together, the different results in terms of disease susceptibility mediated by the *IL18 *gene polymorphisms in different autoimmune diseases support the notion that different pathogenic mechanisms are involved in the development of polygenic diseases.

Although our data show a clear association of these polymorphisms with GCA susceptibility in the Spanish population, further studies in other populations with different genetic backgrounds are needed to clarify fully the implication of *IL18 *promoter polymorphisms in GCA susceptibility. However, most genetic associations reported in Spanish patients with GCA also have been replicated in other populations, such as *HLA-DRB1 *in North American [16,17], Danish [21], French [60], and Swiss [61], and *IL-6 *promoter and eNOS polymorphisms, in Italians [24,28]. This evidence may indicate a high reproducibility of the genetic associations with GCA among different populations and that the potential association with *IL18 *may be also found in other populations. Nevertheless, the lack of genome-wide association studies or whole-genome-scan linkage studies in GCA makes necessary an independent replication study to confirm our results by using a population of a different genetic background.

When we determined the joint effect of the risk alleles of *IL18 *and *TLR4*, we observed a considerably increased risk of GCA (OR, 3.7) for those 25% GCA patients who carried three or more risk alleles compared with those who carried none. Interestingly, this OR was higher than that obtained for any *IL18 *or *TLR4 *SNPs individually (OR, 1.37 for *IL18 *-607; OR, 1.48 for *IL18 *-1297, and OR, 1.65 for *TLR4 *+896 G allele). The additive effect observed between *IL18 *and *TLR4 *suggests that combining information from common risk polymorphisms could improve disease prediction. These observations, as well as the findings showing that *IL18 *and *TLR4 *genetic variants are associated with other autoimmune diseases [33,39-41,50,52,62-64], support the pivotal role of innate immunity in the development of autoimmunity and GCA. Nevertheless, further studies in other populations are required to validate our findings.

## Conclusions

The present study shows for the first time that *IL18 *gene promoter polymorphisms are associated with susceptibility to biopsy-proven GCA. In addition, an additive effect between the risk *IL18 *and *TLR4 *alleles was observed.

## Abbreviations

CI: confidence interval; GCA: giant cell arteritis; IL18: interleukin 18; OR: odds ratio; SNP: single-nucleotide polymorphism; TLR4: Toll-like receptor 4.

## Competing interests

The authors declare that they have no competing interests.

## Authors' contributions

RPM carried out genotyping, participated in the design of the study, data analysis, and helped to draft the manuscript. TRV participated in the acquisition and interpretation of data and in the design of the study. OT participated in the acquisition and interpretation of data. ICM participated in the acquisition and interpretation of data. SC has been involved in the acquisition and interpretation of data and in revising it critically for important intellectual content. JAM participated in the acquisition and interpretation of data. JLC participated in the acquisition and interpretation of data. BF has been involved in the acquisition and interpretation of data and in revising it critically for important intellectual content. MAG-G made substantial contributions to the conception and design of the study, acquisition of data, coordination, helped to draft the manuscript, and gave final approval of the version to be published. JM made substantial contributions to the conception and design of the study, acquisition of data, and coordination, helped to draft the manuscript, and gave final approval of the version to be published.

## Supplementary Material

Additional file 1 **Supplementary table**. Distribution of *IL18/TLR4 *genotype combinations in GCA patients and controls. Click here for file
