# Supplementary material for: Association between IL-18 gene polymorphisms and biopsy-proven giant cell arteritis
Source: Arthritis Res Ther. 2010 Mar 23;12(2):R51. doi: 10.1186/ar2962 (PMC2888200; doi:10.1186/ar2962)
Supplement: Additional file 1 — Supplementary table. Distribution of IL18/TLR4 genotype combinations in GCA patients and controls. [file ar2962-S1.DOC]

| **Table 2.** Distribution of *IL18/TLR4* genotype combinations in GCA patients and controls. | | | | | | | | | | | |
| --- | --- | --- | --- | --- | --- | --- | --- | --- | --- | --- | --- |
|  | | | | | | | | | | | |
| Number of | GCA^b^ | | Controls^b^ | |  | | Genotype | | |  | |
| risk alleles^a^ | Counts | Frequency  (%) | Counts | Frequency  (%) | *IL18* –607 (C->A) | | | *IL18* –1297 (T->C) | *TLR4* Asp299Gly |  | |
|  |  |  |  |  |  | | |  |  |  | |
| 5 | 0 | 0 | 2 | 0.84 | AA | | | CC | AG |  | |
| 4 | 6 | 3.0 | 10 | 4.2 | AA | | | CC | AA |  | |
| 4 | 2 | 1.0 | 1 | 0.4 | AA | | | CT | AG |  | |
| 4 | 1 | 0.5 | 0 | 0 | AC | | | CC | AG |  | |
| 3 | 1 | 0.5 | 0 | 0 | AA | | | TT | AG |  | |
| 3 | 23 | 11.9 | 7 | 2.9 | AA | | | CT | AA |  | |
| 3 | 4 | 2.0 | 3 | 1.26 | AC | | | CC | AA |  | |
| 3 | 13 | 6.5 | 8 | 3.3 | AC | | | CT | AG |  | |
| 3 | 0 | 0 | 0 | 0 | CC | | | CC | AG |  | |
| 2 | 43 | 21.9 | 69 | 29.0 | AC | | | CT | AA |  | |
| 2 | 8 | 4.0 | 9 | 3.8 | AC | | | TT | AG |  | |
| 2 | 4 | 2.0 | 0 | 0 | CC | | | CC | AA |  | |
| 2 | 1 | 0.5 | 2 | 0.8 | CC | | | CT | AG |  | |
| 2 | 8 | 4.0 | 4 | 1.7 | AA | | | TT | AA |  | |
| 1 | 39 | 19.9 | 37 | 15.5 | AC | | | TT | AA |  | |
| 1 | 8 | 4.0 | 4 | 1.7 | | CC | | CT | AA |  | |
| 1 | 7 | 3.5 | 13 | 5.5 | | CC | | TT | AG |  | |
| 0 | 29 | 14.9 | 66 | 27.7 | | CC | | TT | AA |  | |
|  |  |  |  |  | |  | |  |  |  | |
|  | | | | | | | | | | |  |

^a^The considered risk alleles are as follows: the A allele of the *IL18* –607; the C allele of the *IL18* –1297 and the G allele of the *TLR4* Asp299Gly.

^b^Individuals with 100% genotype success rate for the three polymorphisms under study.
